# A Comparison of XGBoost, Random Forest, and Nomograph for the Prediction of Disease Severity in Patients With COVID-19 Pneumonia: Implications of Cytokine and Immune Cell Profile

**DOI:** 10.3389/fcimb.2022.819267

**Published:** 2022-04-12

**Authors:** Wandong Hong, Xiaoying Zhou, Shengchun Jin, Yajing Lu, Jingyi Pan, Qingyi Lin, Shaopeng Yang, Tingting Xu, Zarrin Basharat, Maddalena Zippi, Sirio Fiorino, Vladislav Tsukanov, Simon Stock, Alfonso Grottesi, Qin Chen, Jingye Pan

**Affiliations:** ^1^ Department of Gastroenterology and Hepatology, The First Affiliated Hospital of Wenzhou Medical University, Wenzhou, China; ^2^ School of the First Clinical Medical Sciences, Wenzhou Medical University, Wenzhou, China; ^3^ Jamil-ur-Rahman Center for Genome Research, Dr. Panjwani Centre for Molecular Medicine and Drug Research, International Center for Chemical and Biological Sciences, University of Karachi, Karachi, Pakistan; ^4^ Unit of Gastroenterology and Digestive Endoscopy, Sandro Pertini Hospital, Rome, Italy; ^5^ Internal Medicine Unit, Budrio Hospital, Bologna, Italy; ^6^ Department of Gastroenterology, Scientific Research Institute of Medical Problems of the North, Krasnoyarsk, Russia; ^7^ Department of Surgery, World Mate Emergency Hospital, Battambang, Cambodia; ^8^ Unit of General Surgery, Sandro Pertini Hospital, Rome, Italy; ^9^ Department of Intensive Care Unit, The First Affiliated Hospital of Wenzhou Medical University, Wenzhou, China

**Keywords:** COVID-19, infection, pneumonia, severity, critically ill, predictor, machine learning

## Abstract

**Background and Aims:**

The aim of this study was to apply machine learning models and a nomogram to differentiate critically ill from non-critically ill COVID-19 pneumonia patients.

**Methods:**

Clinical symptoms and signs, laboratory parameters, cytokine profile, and immune cellular data of 63 COVID-19 pneumonia patients were retrospectively reviewed. Outcomes were followed up until Mar 12, 2020. A logistic regression function (LR model), Random Forest, and XGBoost models were developed. The performance of these models was measured by area under receiver operating characteristic curve (AUC) analysis.

**Results:**

Univariate analysis revealed that there was a difference between critically and non-critically ill patients with respect to levels of interleukin-6, interleukin-10, T cells, CD4^+^ T, and CD8^+^ T cells. Interleukin-10 with an AUC of 0.86 was most useful predictor of critically ill patients with COVID-19 pneumonia. Ten variables (respiratory rate, neutrophil counts, aspartate transaminase, albumin, serum procalcitonin, D-dimer and B-type natriuretic peptide, CD4^+^ T cells, interleukin-6 and interleukin-10) were used as candidate predictors for LR model, Random Forest (RF) and XGBoost model application. The coefficients from LR model were utilized to build a nomogram. RF and XGBoost methods suggested that Interleukin-10 and interleukin-6 were the most important variables for severity of illness prediction. The mean AUC for LR, RF, and XGBoost model were 0.91, 0.89, and 0.93 respectively (in two-fold cross-validation). Individualized prediction by XGBoost model was explained by local interpretable model-agnostic explanations (LIME) plot.

**Conclusions:**

XGBoost exhibited the highest discriminatory performance for prediction of critically ill patients with COVID-19 pneumonia. It is inferred that the nomogram and visualized interpretation with LIME plot could be useful in the clinical setting. Additionally, interleukin-10 could serve as a useful predictor of critically ill patients with COVID-19 pneumonia.

## Highlights

1. XGBoost exhibited the highest discriminatory performance for prediction of critically ill patients with COVID-19 pneumonia.
2. The nomogram and visualized interpretation with LIME plot could be useful in the clinical setting.
3. Interleukin-10 is a useful predictor of critically ill patients with COVID-19 pneumonia.

## Introduction

Coronavirus disease 2019 (COVID-19) is a newly recognized illness, caused by the highly contagious severe acute respiratory syndrome coronavirus 2 (SARS-CoV-2) and spread rapidly around the world in the last two years (Hong et al., 2021). As of February 28, 2022 (based on the WHO statistics), over 430 million confirmed cases and over 5.7 million deaths have been recorded (2022). COVID-19 causes a spectrum of symptoms ranging from mild to severe pneumonia as well as asymptomatic cases. Our previous study indicated that 34.9% patients with viral pneumonia would develop critical illness, and required admission to the ICU. They either had a fraction of inspired oxygen (FiO2) value of at least 60% or more during hospitalization and required mechanical ventilation (Hong et al., 2021). Delayed presentation of symptoms increases the risk of mortality and need for high-intensity healthcare (Suliman et al., 2021). The 28-day mortality span was reported for 61.5% of critically ill patients, with an average interval of 7 days between ICU admission to death in Wuhan, China (Yang et al., 2020). Early identification of critical illness grants an opportunity for timely intervention and thus, prevent more complicated, protracted and less successful hospital admissions (Suliman et al., 2021).

Anurag et al. validated the Pneumonia Severity Index (PSI)/PORT, Confusion, Respiratory rate, Blood pressure, 65 years of age and older (CURB-65) and the Severe Community-Acquired Pneumonia (SCAP) scoring system in COVID-19 pneumonia, for prediction of disease severity and 14-day mortality (Anurag and Preetam, 2021). However, in this study the severe COVID-19 pneumonia was defined by PSI/PORT score >130, CURB-65 score ≥53 or SCAP score ≥10 (Anurag and Preetam, 2021). San et al. classified the disease severity according to the interim guidance of the World Health Organization (San et al., 2021). They suggested that predicting high-risk group by the Brescia-COVID Respiratory Severity Scale (BRCSS) and quick SOFA (qSOFA), may improve clinical outcomes in COVID-19 patients (San et al., 2021). Bats et al. defined the severity with arterial oxygen saturation (SaO2) of less than 90% on room air or need of ≥4 L/min oxygen therapy (O2) to obtain a SaO2 ≥94% (Bats et al., 2021) and developed a COVID-19 severity risk score upon hospital admission (Bats et al., 2021). By enrolling patients both with and without pneumonia and using the definition of severity of COVID-19 recommended by the National Health Commission of China, Liang et al. developed a clinical risk score to predict the occurrence of critical illness in hospitalized patients with COVID-19 (Liang et al., 2020a). Using the same definition of severity of COVID-19 as Liang et al. (2020a), Zhang et al. (2020) developed a score consisting of age, WBCs, neutrophil count, glomerular filtration rate and myoglobin, for prediction of disease severity in COVID-19 (Zhang et al., 2020). Nomogram is a mathematical model that allows for individualized and evidence-based risk estimation, facilitating management-based decision-making. Feng et al. divided patients into three types (moderate, severe, and critical type) and reported that a nomogram based on chest CT and clinical characteristics could predict the disease progression in COVID-19 pneumonia patients much earlier (Feng et al., 2020). Li reported that, by using the definition of severity of COVID-19 recommended by the National Health Commission of China, a nomogram consisting of CT-based radiomics signature could be used for predicting severe COVID-19 pneumonia (Li et al., 2021). It has already been applied in COVID-19 to predict mortality (Ji et al., 2020) and assess survival (Dong et al., 2021). In summary, different studies used different definition of severity of disease and inclusion criteria. Few included clinical and laboratory prediction scores to identify critical illness in patients with CT confirmed COVID-19 pneumonia.

Machine learning (ML) methods such as deep learning, extreme gradient boosting (XGBoost) and RF focus on how computers learn from data before being applied to real settings. ML methods are useful for developing robust risk models and redefining patient classes (Deo, 2015). Therefore, many applications of ML exist (for clinical diagnosis, prediction, and classification of patients with COVID-19) (Mottaqi et al., 2021). Previously, Liang et al. have developed a deep learning mediated survival Cox model for early triage of critically ill COVID-19 patients (with and without X-ray abnormality) (Liang et al., 2020b). Deep Learning has also been used for the predictive model for the identification of natural molecules as potential inhibitors of SARS-CoV-2 inhibitors of main protease (Joshi et al., 2021). The XGBoost algorithm has shown to outperform other techniques for various sets of features, in a variety of different settings. Yan et al. has used XGBoost algorithm to identify lactic dehydrogenase (LDH), lymphocyte and C-reactive protein (CRP) as predictors of the mortality of individual patients (Yan et al., 2020). Wang et al. applied the XGBoost model to build a mortality-prediction model using clinical and laboratory data parameters for extrapolation of in-hospital mortality in patients with COVID-19 (Wang et al., 2020b). Liu et al. developed an XGBoost-based clinical model consisting of lymphocyte percentage, lactic dehydrogenase, neutrophil count, and D-dimer on admission for predicting critical illness risk in hospitalized patients with COVID-19 pneumonia (Liu et al., 2021). Ryan et al. reported that XGBoost-based algorithm is a useful predictive tool for anticipating patient mortality in COVID-19, pneumonia, and mechanically ventilated patients (Ryan et al., 2020).

In addition, most of the existing scores are developed based on only clinical and laboratory features. As the severity of COVID-19 pneumonia is clearly associated with multifactorial responses, the use of only clinical and laboratory features may result in missing important information from other risk factors. Cytokine storm plays an important role in severe COVID-19 pneumonia (Hu et al., 2021). Therefore, in most severe cases, the prognosis can be markedly worsened by the hyperproduction of proinflammatory cytokines, such as Interleukin-6 (IL-6) and TNF-α, preferentially targeting lung tissue (Costela-Ruiz et al., 2020). However, immune cells such as B Lymphocytes, T cells, CD4^+^ T & CD8^+^ T cells, and cytokine profile (such as IL-10), are rarely enrolled in these scores. Hence, further studies are required for developing scoring systems for prediction of critically ill patients with COVID-19 pneumonia (with both cytokine profiles and immune cell data).

The first aim of this study was to develop and compare an extreme gradient boosting (XGBoost) model, RF model, and a conventional LR model (present as nomogram) based on clinical, laboratory data and immune cells and cytokine profiles for prediction of critically ill patients with COVID-19 pneumonia. The second aim was to evaluate the role of immune cells and cytokine profile as potential predictors of the severity of COVID-19 pneumonia.

## Material and Methods

### Study Design, Subject Selection and Ethics

We conducted a *post-hoc* analysis of a previously reported retrospective cohort study in the First Affiliated Hospital of Wenzhou Medical University in mainland China (Hong et al., 2021). All patients with confirmed COVID-19 pneumonia between January 2020 and March 2020 were eligible for inclusion in this study. A confirmed case of COVID-19 was defined as a positive result on a real-time reverse-transcriptase–polymerase-chain-reaction (RT-PCR) assay of nasal and pharyngeal swab specimens (Hong et al., 2021). Exclusion criteria included lack of pneumonia and unavailability of chest computed tomography scans.

### Definition of Severity

Patients with COVID-19 pneumonia were defined as critically ill if they were admitted to the intensive care unit (ICU) and required mechanical ventilation or had a fraction of inspired oxygen (FiO2) of at least 60% or more (Kumar et al., 2009; Hong et al., 2021).

### Data Collection and Follow Up

The epidemiological, clinical symptoms & signs, laboratory parameters, cytokine profile, and immune cell data on admission were obtained using data collection forms of electronic medical records. These data included blood chemical analysis, liver, and renal function testing, glucose and coagulation testing, creatine kinase, B-type natriuretic peptide, C-reaction protein, procalcitonin, IL-2, IL-4, IL-6, IL-10, and tumor necrosis factor (TNF)-α, B Lymphocytes, T cells, CD4^+^ T and CD8^+^ T cell count. All patients were followed up until March 12, 2020 (Hong et al., 2021). We used LR and machine learning models to differentiate critically ill from non-critically ill patients with COVID-19 pneumonia.

### Statistical Analysis

There were missing values in D-dimer, B-type natriuretic peptide levels, cytokine profiles, and immune cells. To handle this issue, missing values were imputed using Multiple Imputations by Chained Equations (MICE), when performing LR and ML analysis (Royston, 2005). MICE has emerged as one of the principal statistical approaches for dealing with missing data. The missing values were replaced by the estimated plausible values to create a “complete” dataset (Royston, 2005).

Categorical values were described as count and proportions and compared by the χ^2^ test or Fisher’s exact test (Hong et al., 2020). According to the results of Shapiro–Wilk test, continuous values were expressed by mean ± SD or median and Inter Quartile Range (IQR) and compared using Student’s t-test or the Wilcoxon non-parametric test. All the variables, found to be different between critically ill and non-critically ill patients on univariate analysis, underwent receiver operating characteristic (ROC) curve analysis to identify the valuable single index predictor of critically ill patients with COVID-19 pneumonia. Then, only variables with the area under the receiver operating characteristic curve (AUC) >0.7 were used as potential predictors for critically ill patients having COVID-19 pneumonia (Hong et al., 2017). In addition, an exploratory variable importance analysis was also performed using both XGBoost and RF method to evaluate the role of different variables in prediction of critical illness. In XGBoost method, SHapley Additive exPlanations (SHAP) summary plot was used to quantify the variable importance of each variable, and SHAP force plot was used to explain the individual predictions, respectively (Deshmukh and Merchant, 2020). In the RF method, the importance of each variable was subsequently measured by calculating how much reduction each variable offers when they were added to the RF model using mean decreased accuracy and Gini (Gong et al., 2020).

Risk models were developed using conventional statistical method (forward-conditional step-wise LR), traditional machine learning algorithm (RF), and current state-of-the-art boosting algorithm utilized for gradient boosted decision trees (XGBoost). An RF model is a collection of many decision tree models, each of which is characterized by a tree-like structure (Gong et al., 2020). A gradient boosting ML algorithm (XGBoost) was employed for a binary classification task based on the presence or absence of critically ill patients with COVID-19 pneumonia (Al’aref et al., 2020).

We randomly held out two patients for individualized prediction, the remainder number (61 patients) was used to develop prediction models. When building and tuning prediction models, we used two-fold cross-validation as the resampling strategy to avoid overfitting of the model on new data. Training set was divided into two equal-sized sub-samples in which one sub-sample was taken for training and the other one for testing over all possible permutations. Analysis was repeated two times (folds). The AUC was calculated for each of the two analyses, using only the respective test data. At last, the mean AUC with 95% CI, and also area under precision recall curve and area under precision recall gain curve was calculated and compared (Saito and Rehmsmeier, 2015). Since the incidence of critical illness in patients with COVID-19 pneumonia was high (34.9%), we selected the best cut-of point (detected where the number of true positives was the highest with sensitivity >90%). This was done by selecting a threshold value at a point where the longest increase in the specificity of the slope declines. The sensitivity, specificity, accuracy, as well as F-score, which is a harmonic mean of recall and precision, were also calculated and compared (Saito and Rehmsmeier, 2015). To overcome the black box problem of XGBoost output and improve its interpretability, the LIME plot was used to explain the individualized prediction.

As for LR analysis, the conditional probabilities for stepwise entry and removal of a factor were 0.05 and 0.06, respectively (Hong et al., 2019). Based on the results of LR, an equation model and nomogram were developed to predict critical illness associated with COVID-19 pneumonia. Model calibration was done by Hosmer–Lomeshow goodness of fit test. Odds ratios (OR) were calculated, with 95% CI. Multicollinearity was considered to be significant if the largest variance inflation factor exceeded 10 (Hong et al., 2020).

A two-tailed P-value of less than 0.05 was considered statistically significant. All statistical analyses were performed in the R and STATA software. Data flow diagram of our study is shown in **Supplementary Figure 1**.

## Results

### Clinical Characteristics

A total of 63 hospitalized patients with confirmed COVID-19 pneumonia were enrolled in this study. Baseline clinical and laboratory findings of all patients on admission have been described before (Hong et al., 2021). In summary, out of the 63 patients, 22 (34.9%) required high-flow nasal cannula or higher-level oxygen support measures to correct hypoxemia during their hospital stay and were classified as critically ill patients. The remaining 41 patients were identified as non-critically ill. The mean age of the patients was 55.9 ± 15.3 years. Among these, 41 (65.1%) patients were men. The mean time from onset of symptoms to the hospital admission was 6.9 ± 3.7 days. The most frequent symptoms at the onset of illness were fever and cough (98.4 and 61.94% respectively). Of the clinical characteristics and laboratory findings, the respiratory rate, leukocyte, neutrophil counts, levels of aspartate transaminase, albumin, serum procalcitonin, D-dimer, and B-type natriuretic peptide levels were useful predictors of critically ill patients with COVID-19 pneumonia, having an AUC of more than 0.7 (Hong et al., 2021). Most patients had increased IL-6, IL-10, and decreased CD4^+^ T cells. The median values of these variables in all patients are shown in the **Table 1**.

**Table 1:** Baseline characteristics of studied variables in the patients (on admission).

Characteristic	Values
Median age, years (IQR)	56 ± 15
Male sex, N (%)	41 (65.1)
Respiratory rate	20 (20–23)
**Laboratory findings**	
Leukocyte (10^9^/L)	6.69 (4.78–11.09)
Neutrophil (10^9^/L)	4.91 (3.1–9.07)
Aspartate transaminase, U/L	32 (25–51)
Albumin, (mg/dl)	33.2 ± 4.6
D-dimer (N = 58), mg/L	0.81 (0.5–1.18)
B-type natriuretic peptide (N = 62), pg/ml	37 (10–66)
Procalcitonin, ng/ml	0.06 (0.04–0.11)
**Cytokine profile**	
IL-2 (N = 45), pg/ml	0.86 (0.64–1.02)
IL-4 (N = 45), pg/ml	0.77 (0.52–1.28)
IL-6 (N = 46), pg/ml	24.44 (5.24–66.9)
IL-10 (N = 45), pg/ml	7.11 (4.43–11.71)
tumor necrosis factor (TNF)-α (N = 46), pg/ml	0.24 (0.1–0.52)
Immune cells	
B Lymphocytes (N = 44), (/ul)	180.5 (117.5–240.5)
T cells (N = 44), (/ul)	452.5 (282.5–653.5)
CD4^+^ T (N = 44), (/ul)	266 (166–387.5)
CD8^+^ T cells (N = 44) (/ul)	152 (93.5–266)

Data are shown either as number of observations, percentage, or median and interquartile range.

### Cytokine and Immune Cells

**Figure 1:**
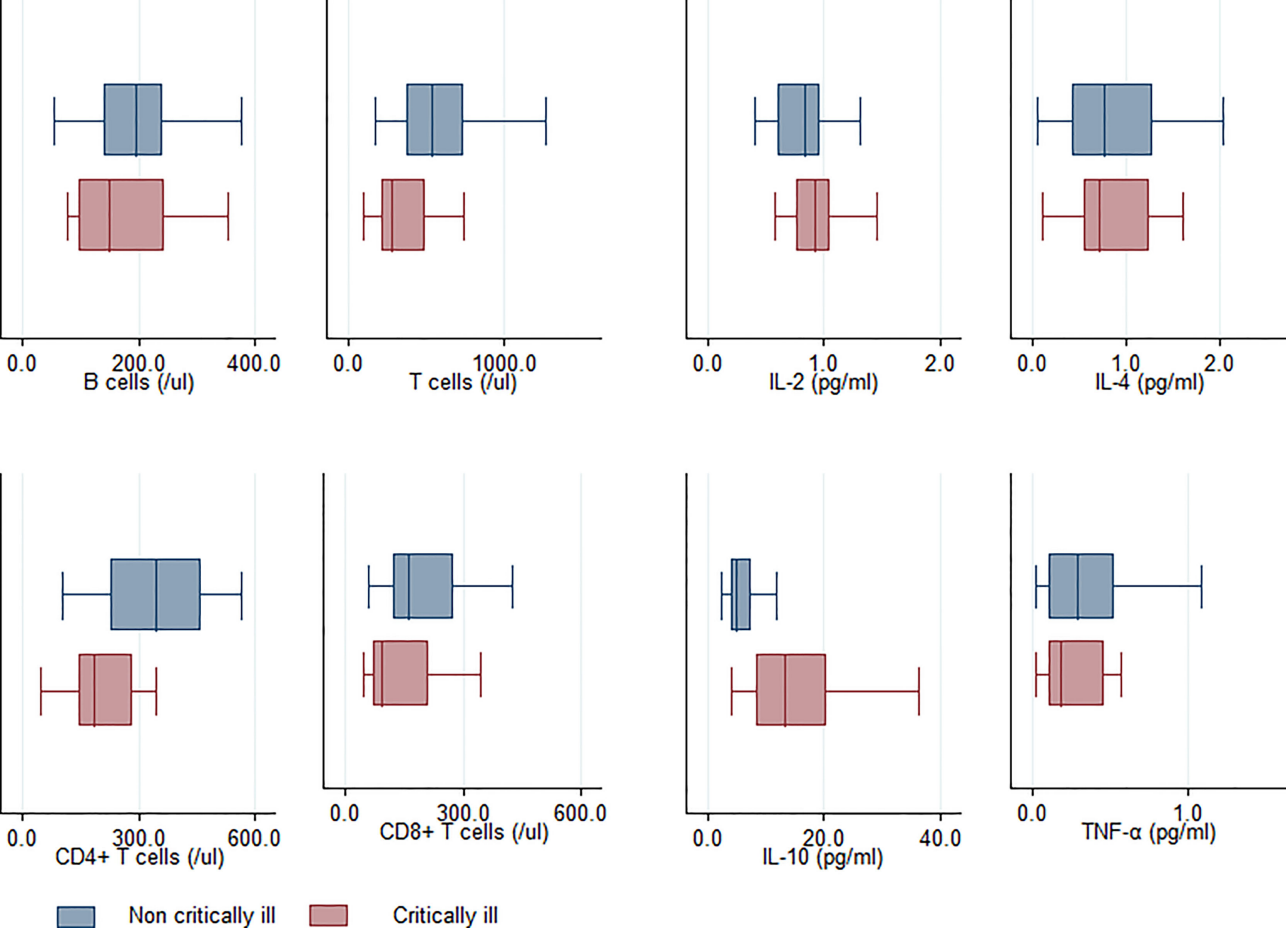
Comparison of cytokine profile and immune cells between critically and non-critically ill patients exhibiting COVID-19 pneumonia.

**Figure 2:**
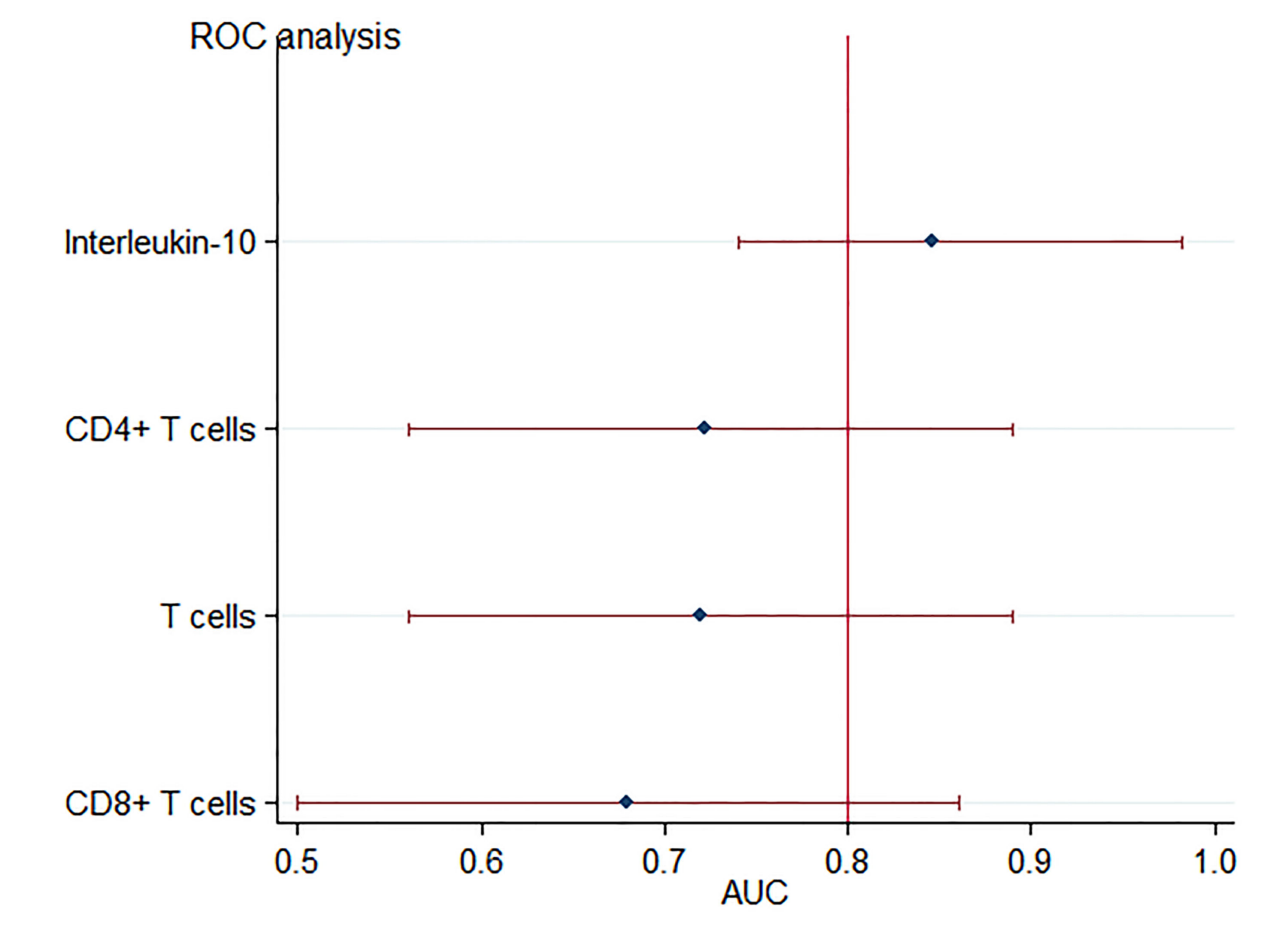
Forest plot for accuracy of IL-10 and T cells in predicting critical illness relate dto COVID-19 pneumonia. Each marker is plotted as an area under the curve (AUC) of the receiver operating characteristic curve, with a 95% confidence interval.

As for the cytokine profiles and immune cells, univariate analysis revealed that in comparison to the non-critically ill patients, patients with critical illness had higher levels of, IL-6 and IL-10, as well as lower levels of T cells, CD4^+^ T, and CD8^+^ T cells (**Figure 1**) (Hong et al., 2021). There was no significant difference observed among patients with respect to IL-2, IL-4, Tumor Necrosis Factor Alpha (TNF-a), and B Lymphocytes. Among these, the T cells (AUC: 0.72 ± 0.09), CD4^+^ T levels (AUC: 0.72 ± 0.08), IL-6 (AUC: 0.85 ± 0.06), and IL-10 (AUC: 0.86 ± 0.06) were useful predictors of critically ill patients with COVID-19 pneumonia, with AUC of more than 0.7 (**Figure 2**) **(** Hong et al., 2021 **)**.

### Exploratory Variable Importance Analysis

**Figure 3:**
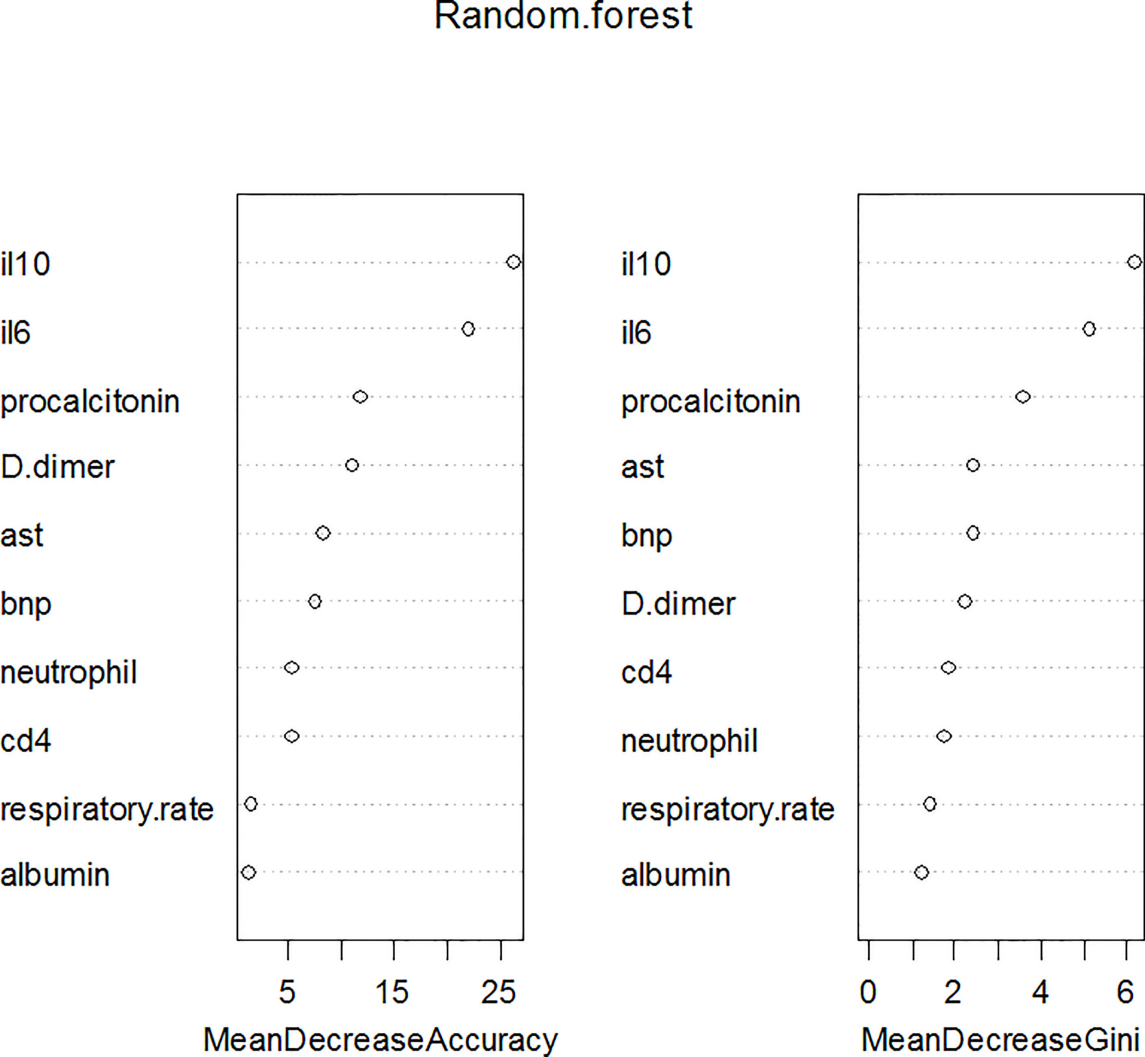
Variable importance plot using RF model for the critically ill COVID-19 pneumonia patients. IL-10 and IL-6 were the most important variables in determining critical illness by either mean decrease accuracy or by mean decrease Gini.

**Figure 4:**
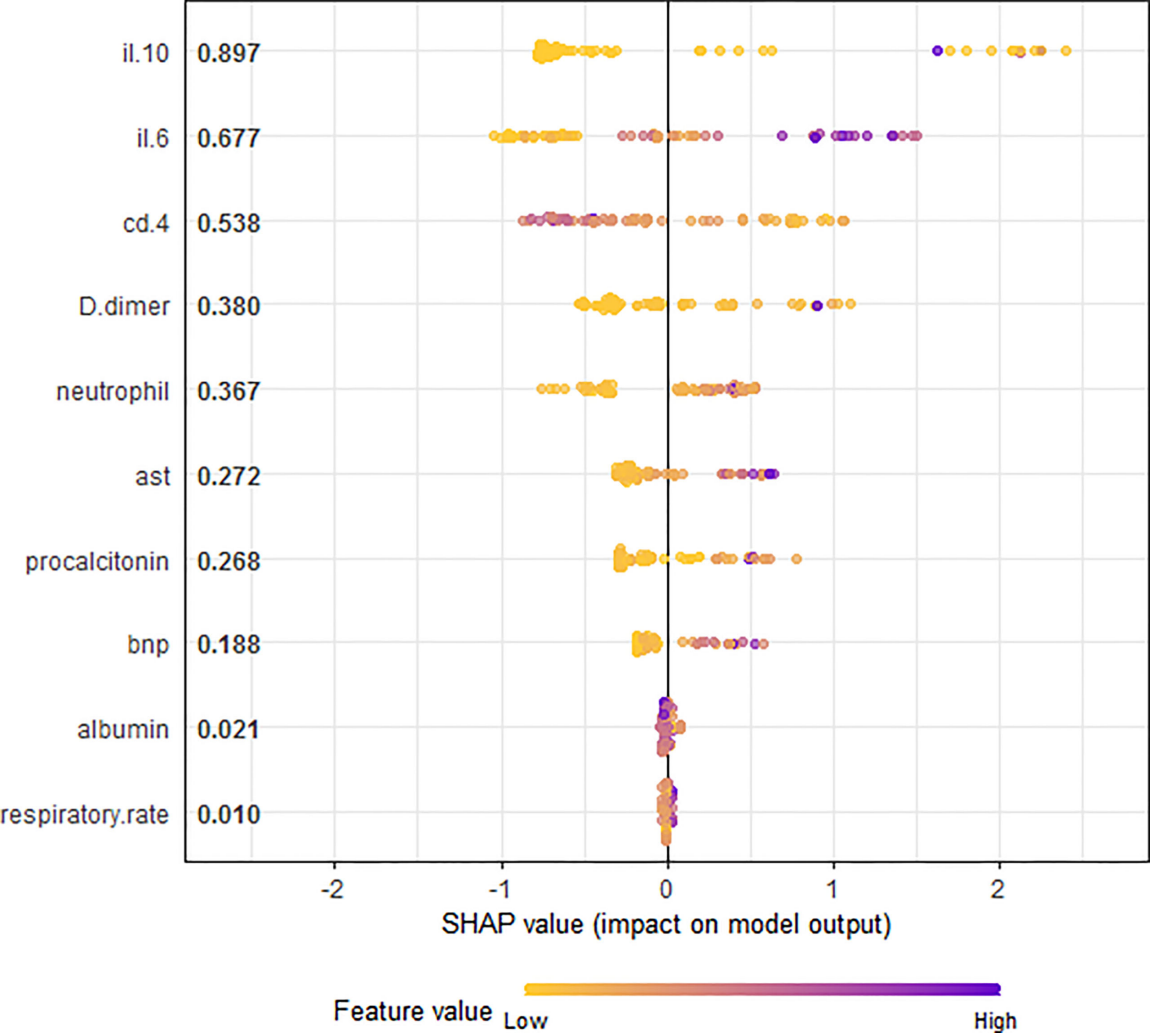
SHAP summary plot for the all the variables contributing to the XGBoost model prediction for critically ill COVID-19 pneumonia patients. This shows the ranking features and their impact on the model output. The horizontal axis shows the corresponding SHAP value of the feature. A positive SHAP value contributes to the prediction of critically ill COVID-19 pneumonia patients and vice versa.

Leukocyte and T cells were not included in further analysis because of strong multicollinearity. Therefore, the ten variables (respiratory rate, neutrophil counts, aspartate transaminase, albumin, serum procalcitonin, D-dimer and B-type natriuretic peptide, CD4^+^ T cells, IL-6 and IL-10) were used for machine learning models. Based on the RF analysis, IL-10 was the most important predictor of critical illness in patients with COVID-19 pneumonia, followed by IL-6 and serum procalcitonin (**Figure 3**). SHAP summary plot revealed the relative importance of each feature in the XGBoost analysis. IL-10, IL-6, and CD4^+^ T cells were the three most important features (**Figure 4**).

### Development and Comparison of Prediction Models

**Figure 5:**
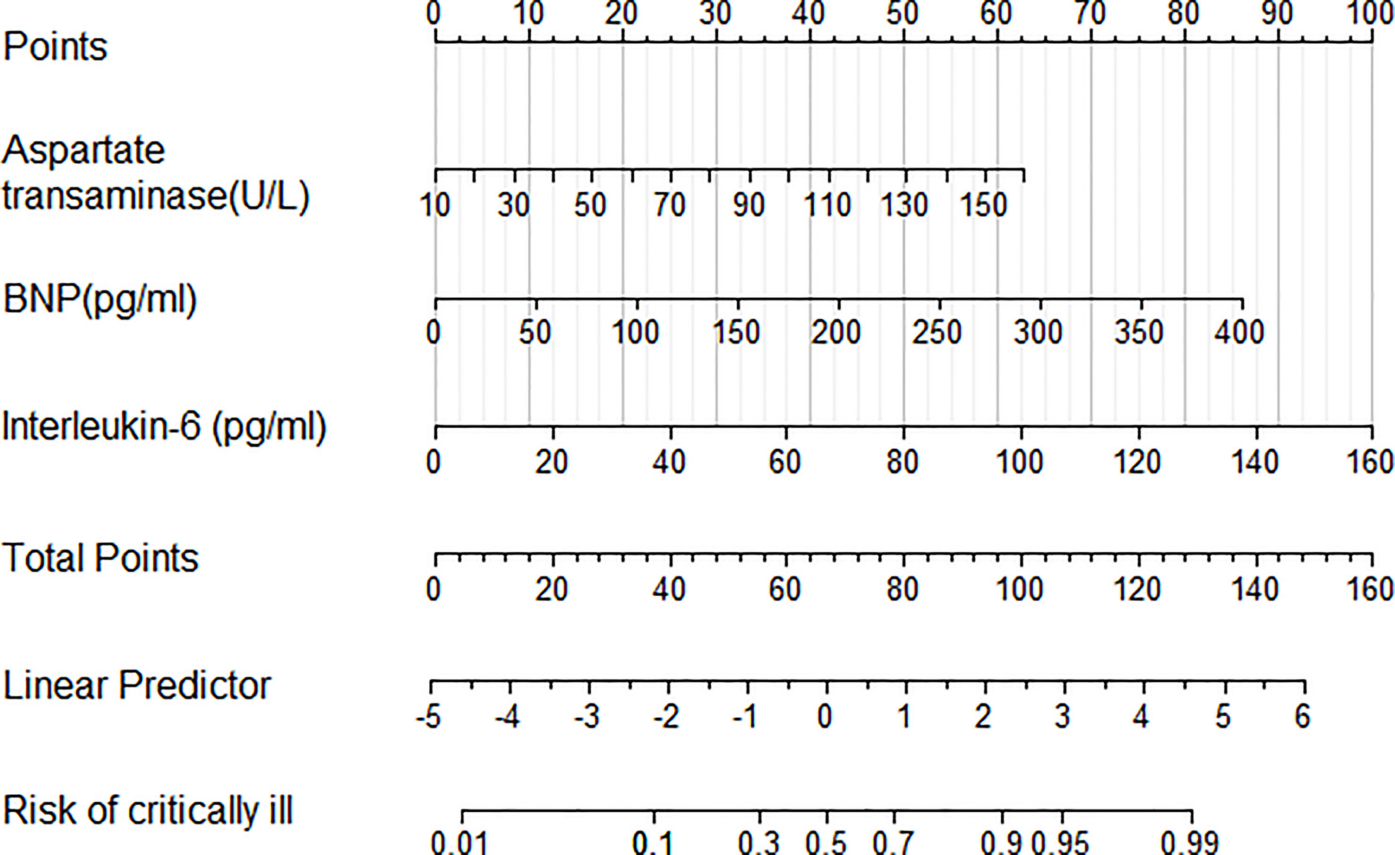
Nomogram predicting the probability of critically ill COVID-19 related pneumonia patients. To obtain the nomogram-predicted probability, patient values have been plotted on each axis. A vertical line to the point axis depicts attributes for each variable value. Summing the points for all variables and obtaining the sum for the point line leads to assessment of the individual probability of critically ill COVID-19 related pneumonia patients.

The same ten variables (respiratory rate, neutrophil counts, aspartate transaminase, albumin, serum procalcitonin, D-dimer, B-type natriuretic peptide, CD4^+^ T cells, IL-6 and IL-10) were used for multivariable logistic analysis. Step-up LR identified the following three independent variables as predictive of critical illness in patients with COVID-19 pneumonia: aspartate transaminase (OR = 1.03, 95% CI 1.01, 1.05, P = 0.026), B-type natriuretic peptide (OR = 1.02, 95% CI 1.01, 1.03, P = 0.011), and IL-6 (OR = 1.04, 95% CI 1.02, 1.06, P <0.001). An LR model was developed to predict critically ill patients with COVID-19 pneumonia as follows: −5.25 + 0.031 aspartate transaminase (U/L) +0.016 B-type natriuretic peptide (pg/ml) +0.046 IL-6 (pg/ml). The coefficients from LR model were utilized to build a nomogram for the prediction of critical illness (**Figure 5**). The Hosmer–Lemeshow goodness-of-fit test was significant (P = 0.4), suggesting that our prediction model fits the actual data well.

**Figure 6:**
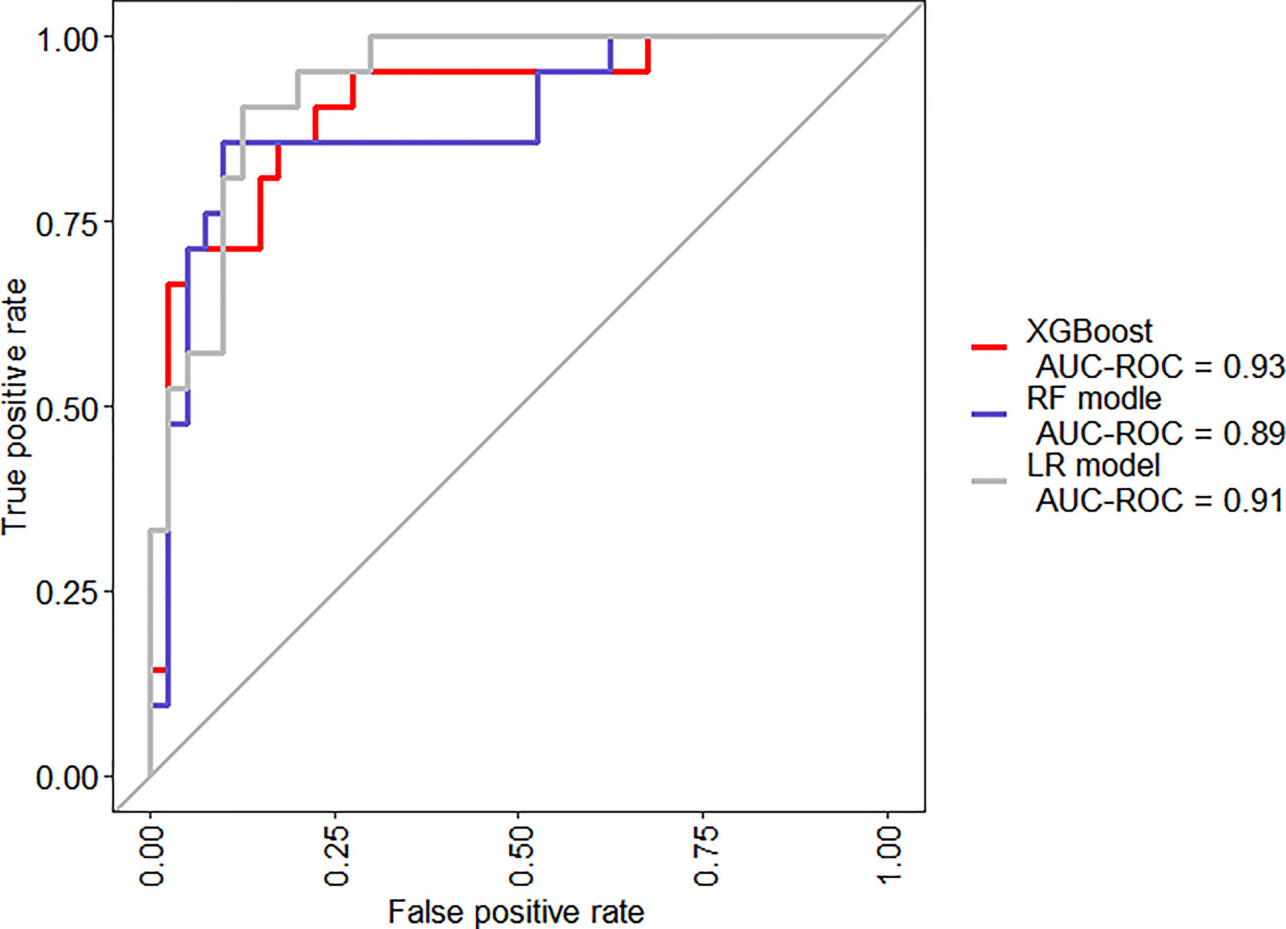
Mean receiver operator characteristic (ROC) curves for the XGBoost, RF model, and LR model.

**Figure 7:**
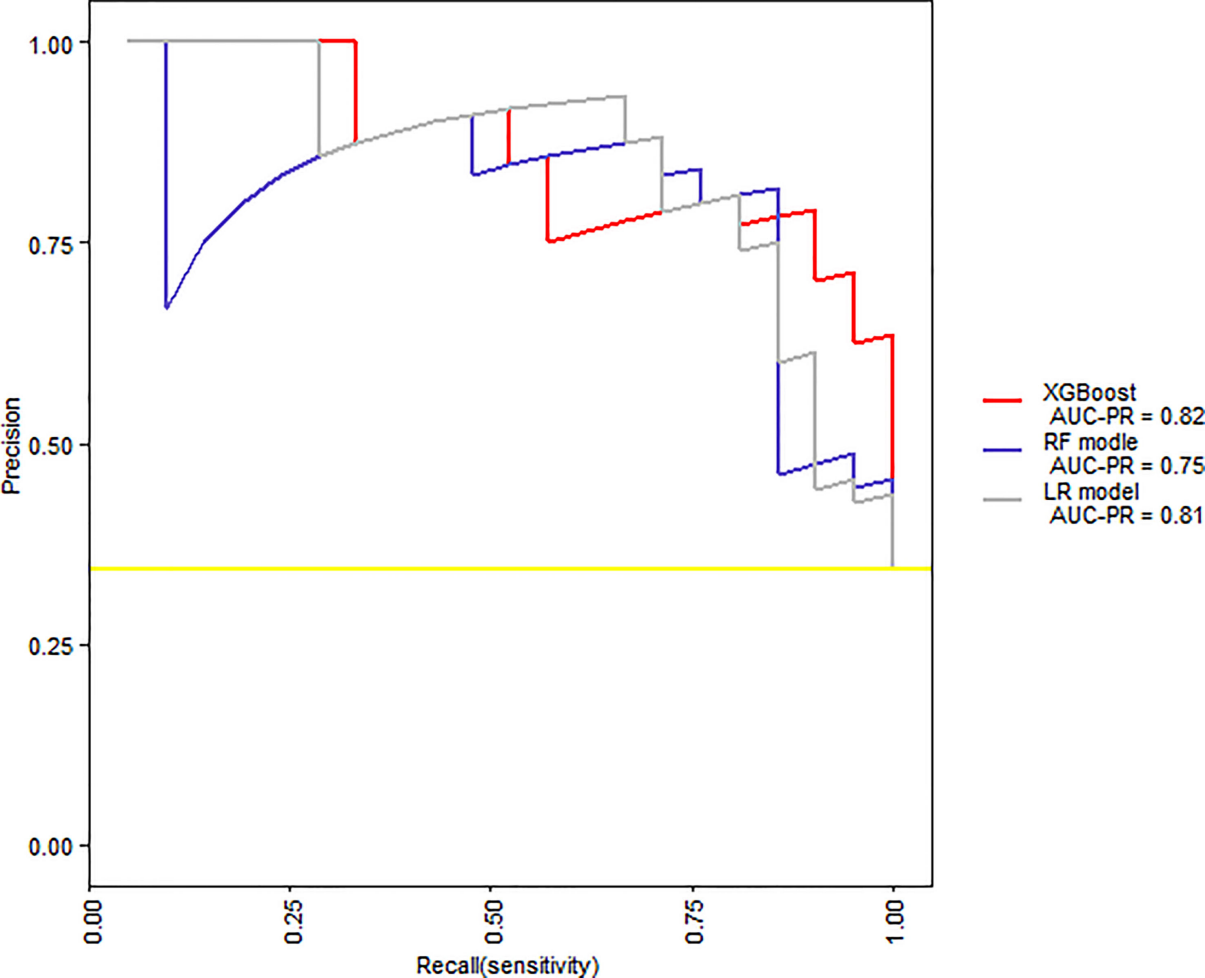
Precision recall curves for the XGBoost, RF model, and LR model.

**Figure 8:**
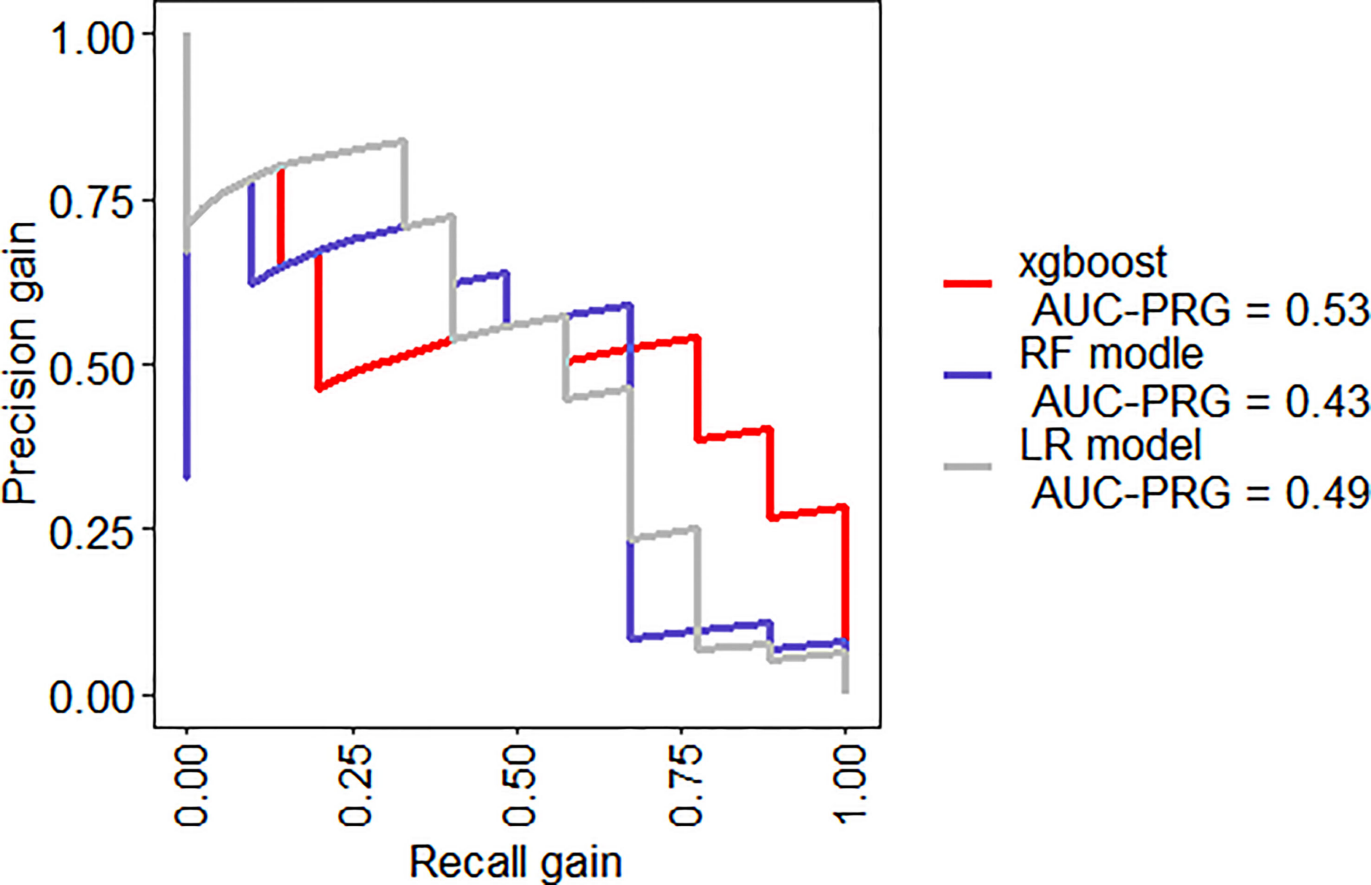
Precision recall gain curves for the XGBoost, RF model, and LR model.

When we compared the predicting models in two-fold cross-validation, the mean AUC of ROC curve analysis for LR model, RF model, and XGBoost model for the prediction of SAP was 0.91, 0.89, and 0.93, respectively (**Figure 6**). The area under precision recall curve also showed that the XGBoost model (0.82) achieved a higher mean area under precision recall curve than that of the LR (0.81) and RF model (0.75) (**Figure 7**). The area under precision recall gain curve for XGBoost model, LR model and RF model was 0.53, 0.49, and 0.43, respectively (**Figure 8**).

XGBoost model achieved a sensitivity of 90.5%, specificity of 87.5%, and diagnostic accuracy of 88.5% and F-score of 84.4%. As a comparison, when RF and LR model achieved a similar of sensitivity of 90.1 and 90.5%, respectively, the RF and LR model achieved a lower specificity, diagnostic accuracy and F-score (**Table 2**).

**Table 2:** Diagnostic values of various models implemented for differentiating critically ill patients with COVID-19 pneumonia.

Variable	AUC	Sensitivity	Specificity	Accuracy	F-score
XGBoost model	0.93	90.5%	87.5%	88.5%	84.4%
RF model	0.89	90.1%	47.5%	62.3%	62.2%
LR model	0.91	90.5%	70.0%	78.8%	73.1%

### Explanation of XGBoost Model Results: Individualized Prediction

**Figure 9:**
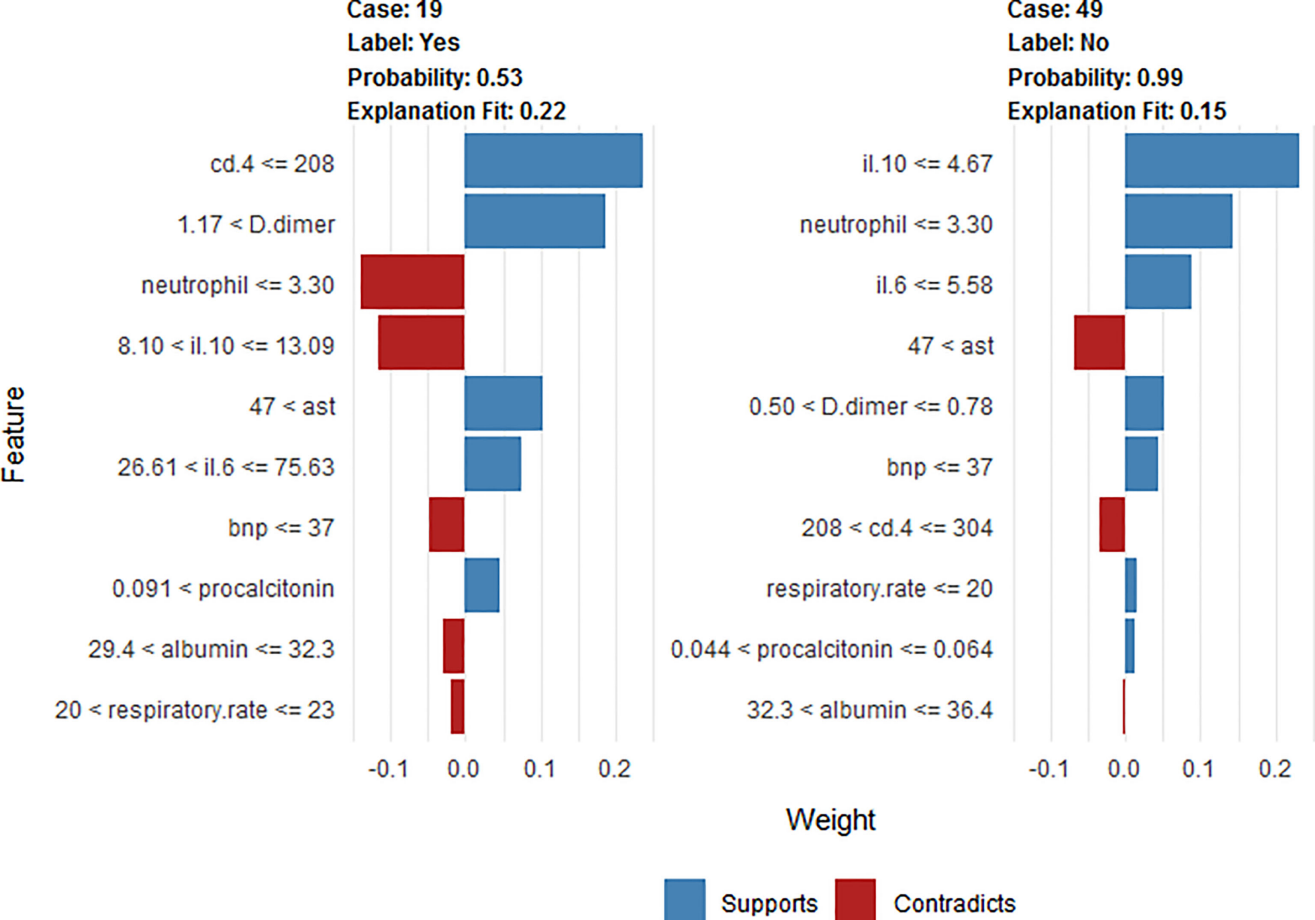
LIME plot explanation of two typical predictions, showing the main contributing features behind the model prediction. The length of the color bar represents the amount of contribution from the corresponding feature.

To clarify the model prediction for individual patients, the LIME plot shows two typical predictions made by the XGBoost model, in which one is for critically ill and the other for non-critically ill patients with COVID-19 pneumonia (**Figure 9**). The length of the bar for each feature indicates the importance (weight) of that feature in making the prediction. A longer bar, therefore, indicates a feature that contributes more towards or against the prediction.

For example, the first case (case 19) is a critically ill patient that was correctly classified. This patient had a respiratory rate of 21 t/min, albumin = 32 mg/dl, Aspartate transaminase = 83 U/L, neutrophil = 2.98 (10^9^/L), Procalcitonin = 0.133 ng/ml, B-type natriuretic peptide = 19 pg/ml, D-dimer = 1.88 mg/L, CD4^+^ T = 167/ul, IL-6 = 74.65 pg/ml, and IL-10 = 10.8 pg/ml. The high IL-6 and decreased CD4^+^ T cells are the main reasons for critical illness factors, outweighing other factors such as normal B-type natriuretic peptide and IL-10.

The second case (case 49) is of a non-critically ill patient with COVID-19 pneumonia classified correctly. This patient had respiratory rate of 12 t/min, albumin = 33.7 mg/dl, Aspartate transaminase = 84 U/L, neutrophil = 2.11(109/L), Procalcitonin = 0.63 ng/ml, B-type natriuretic peptide = 15 pg/ml, D-dimer = 0.72 mg/L, CD4^+^ T = 257/ul, IL-6 = 2.46 pg/ml, and IL-10 = 3.5 pg/ml. The normal IL-10 and IL-6 are the main reasons for non-critical illness factors, outweighing other factors such as decreased albumin.

## Discussion

IL-10 can be produced by many different myeloid and lymphoid cells, especially produced in large quantity by T helper 2 (Th2) during COVID-19 infections (Huang et al., 2020). It serves as an anti-inflammatory cytokine by suppressing macrophage and Dendritic Cells (DCs), thereby limiting T helper 1 (Th1) and T helper 2 (Th2) effector responses (Couper et al., 2008). Premature excretion during a virulent infection can cause overwhelming infection. Conversely, it may lead to severe tissue damage when produced too late during an avirulent infection (Couper et al., 2008). A recent study proposed that dramatic early proinflammatory IL-10 elevation may play a pathological role in COVID-19 severity as its pro-inflammatory or anti-inflammatory effects that distinguish depending on the different course of disease (Lu et al., 2021). Increasing evidence supports the elevation of IL-10 is correlated to the severity of COVID-19 (Han et al., 2020; Huang et al., 2020; Wang et al., 2020a; Zhao et al., 2020; Lu et al., 2021). Our study indicated the importance of IL-6 and 10 variables for RF (**Figure 3**) and SHAP summary plot in XGBoost method (**Figure 4**). Results confirm that IL-10 is the most important variable for the prediction of critical illness in patients with COVID-19 pneumonia. In addition, based on ROC analysis, IL-10 (AUC = 0.86) could be a useful single predictor of critically ill patients with COVID-19 pneumonia (**Figure 2**). The critically ill patients with pneumonia caused by this virosis are those who need high-flow nasal cannula or higher-level oxygen support measures to correct the hypoxemia. They are always observed to have pulmonary fibrotic changes on CT scans, ranging from fibrosis associated with pneumonia to severe lung injury, which results in hypoxemia (Shi et al., 2020). Several *in vivo* and *in vitro* studies have demonstrated that IL-10 demonstrates anti-fibrotic function in pancreatic, liver, and bleomycin-induced lung fibrosis (Thompson et al., 1998; Demols et al., 2002; Shamskhou et al., 2019). Therefore, it is speculated that IL-10 may play an anti-inflammatory and anti-fibrotic role for critically ill patients with COVID-19 pneumonia.

IL-6 is a pleiotropic cytokine secreted by myeloid cells following immune challenge or tissue injury (Yousif et al., 2021). It has a pro-inflammatory function but also has anti-inflammatory, pro-resolution, and regenerative properties (Mcelvaney et al., 2021). Production of IL-6 helps promote resistance to different pathogens and the maintenance of tissue homeostasis, but the overproduction causes chronic inflammatory disorders and severe hyperinflammation (Jones and Hunter, 2021). Several studies have reported that serum level of IL-6 is significantly elevated in the setting of severe COVID-19 disease (Coomes and Haghbayan, 2020; Cummings et al., 2020; Huang et al., 2020; Leisman et al., 2020). Moreover, the use of tocilizumab, a blocker of IL-6 receptor (IL-6R), has been recommended for severe cases of COVID-19 (Huang et al., 2020; Ruan et al., 2020; Wu et al., 2020a; Angriman et al., 2021; Galván-Román et al., 2021; Mcelvaney et al., 2021). IL-6 is also reported as one of the good predictors of progression and severity in patients with COVID-19 (Guirao et al., 2020; Liu et al., 2020; Broman et al., 2021; Ren et al., 2021). In addition, it is suggested that an elevated level of IL-6 is an important predictor of patients with severe COVID-19 needing ventilator support (Galván-Román et al., 2021). Therefore, IL-6 may be an effective marker of both disease severity and decision making in the clinical management of patients. As expected, our study suggests IL-6 (OR = 1.04, 95% CI 1.02, 1.06) is independently associated with critical illness in patients with COVID-19 pneumonia (**Figure 5**).

Aspartate aminotransferase (AST) is one type of aminotransferase that mainly exists in the liver and plays a role in the conversion of aspartate to ketoglutaric acid (Kwo et al., 2017). AST is normally present in the cytoplasm, but it is released into the serum after the damage of cells (Abd Rashid et al., 2021). Therefore, it is used as a method of assessing the liver condition. Recently, studies have reported that critically ill patients with COVID-19 pneumonia manifest elevated AST level (Zahedi et al., 2021). Among indicators of liver injury, elevated AST has been connected with the highest risk of death and the highest association with mortality (Lei et al., 2020). Padmaprakash et al. have demonstrated that AST is a significant predictor of COVID-19 mortality and elevated AST level is a valid indicator of COVID-19 pneumonia severity (Padmaprakash et al., 2022). Elevated AST levels have been independently associated with adverse clinical outcomes in COVID-19 patients, which includes admission to ICU, use of invasive mechanical ventilation, and death (Yip et al., 2021). At admission, AST has been demonstrated as an independent predictor of COVID-19 mortality, and it is essential to monitor AST in hospitalized patients (Ding et al., 2021). As expected, our LR model suggested that AST (OR = 1.03, 95% CI 1.01, 1.05) could be a predictive mark of critically ill patients with COVID-19 pneumonia (**Figure 5**).

Brain natriuretic peptide (BNP) is a 32 amino acid cardiac natriuretic peptide hormone, which is strongly upregulated in cardiac failure and locally in the area surrounding a myocardial infarction (Hall, 2004). Previous studies have highlighted that COVID-19 is a complex disease, targeting many organs and it is an independent risk factor for acute myocardial infarction, promoting the release of BNP (Katsoularis et al., 2021). Emerging data suggest that cardiac injury, manifested by cardiac biomarker elevation, is detected in sizeable COVID-19 patients and is associated with adverse outcomes and increased mortality (Qin et al., 2020). Stefanini et al. suggested that concomitant elevation of both BNP and troponin I serves as a strong independent predictor of all-cause mortality (OR 3.24) (Stefanini et al., 2020). Our study suggested that BNP (OR = 1.02, 95% CI 1.01, 1.03, P = 0.011) was independently associated with the development of critical illness in patients with COVID-19 pneumonia (**Figure 5**).

CD4^+^ T cells are instrumental as activators of both the innate and adaptive arms of the immune system (Ruterbusch et al., 2020). As critical protectors from infectious diseases, they can assist in humoral responses, indirectly activate macrophages, and directly suppress inflammation (Miller and Mitchell, 1969; Parish and Liew, 1972; Jandinski et al., 1976). Rydyznski et al. have suggested that SARS-CoV-2-specific CD4^+^ T cells are strongly associated with COVID-19 disease severity (Rydyznski Moderbacher et al., 2020). Oja et al. reported that CD4^+^ T-cell responses were qualitatively impaired in critically ill patients with COVID-19 patients (Oja et al., 2020). Our study suggested that in comparison to the non-critically ill patients, patients with critical illness had lower levels of CD4^+^ T cells (**Figure 1**). The SHAP summary plot by the XGBoost method suggested that the CD4^+^ T cells play an important role in predicting critical illness (**Figure 4**).

Nomogram is a two-dimensional graphical tool that could be used to predict the probability of a result, consisting of several lines arranged in proportions (Rahman et al., 2021). It demonstrates a great superiority in quantifying the risk of clinical events simply and intuitively (Iasonos et al., 2008; Jin et al., 2017). It is a quantitative and practical prediction tool and could provide clinicians with an easy-to-use method to predict severe pneumonia in COVID-19 patients (Feng et al., 2020). Wu established a nomogram model consisting of seven variables (age, lymphocyte, CRP, LDH, creatine kinase, urea and calcium) for severity risk prediction of COVID-19 pneumonia and classify COVID-19 patients into low-risk, medium-risk, and high-risk groups (Wu et al., 2020b). Incorporating different factors to construct a nomogram could have different clinical values. Ding et al. suggested that the prognosis of COVID-19 patients can be accurately predicted by the nomogram incorporating abnormal AST and D-bilirubin levels along with other individual signs at admission (Ding et al., 2021). Our study suggested that a nomogram based on LR model, consisting of IL-6, AST, and BNP achieved an excellent AUC of 0.91 for prediction of critically ill patients with COVID-19 pneumonia in two-fold cross-validation (**Figure 6**). Compared to other studies (Wu et al., 2020b; Ding et al., 2021), our nomogram was more simple to calculate because only three variables were needed (**Figure 5**). The point of each variable can be determined by referring vertically to the dotted line at the bottom. The scores of each corresponding variable have been added to calculate the total score, and the probability of severe COVID-19 pneumonia is predicted based on the values of the total points and lines, corresponding to the total score.

Compared to other ML methods, XGBoost shows resistance to overfitting in datasets with imbalanced feature/outcome ratios and hyperparameters, which allows tuning for imbalanced datasets (Vaid et al., 2021). By using SHAP summary plot, the variable importance of each variable could be quantified and explained. SHAP values are a game-theoretic approach to model interpretability that provide explanations of global model structure based on combinations of local explanations for each prediction (Vaid et al., 2021). XGBoost has been used to predict respiratory failure within 48 h, morbidity and mortality in patients hospitalized with COVID-19 (Pan et al., 2020; Bolourani et al., 2021; Wang et al., 2021). AlJame et al. (2021) used RF and XGBoost for screening COVID-19 from other patients while Montomoli et al. (2021) has used it to predict change in the SOFA score in a five day span for ICU admitted COVID-19 patients. Feng et al. (2021) used RF and XGBoost for predicting mortality in Covid-19 patients in comparison to several other methods and found XGBoost to be the superior ML method. Iwendi et al. (2020) reported use of RF for COVID-19 mediated deaths with respect to gender, age and geography. They reported more deaths in males, Wuhan population and people aged between 20 and 70 years.

Our study suggested that, when comparing the performance of the XGBoost model with the RF and LR models, the XGBoost (AUC = 0.93) exhibited the highest discriminatory performance, followed by LR (AUC = 0.91) and FR model (AUC = 0.89) (**Figure 6**). The area under precision recall curve and area under precision recall gain curve analysis showed similar results (**Figures 7**, **8**). XGBoost model achieved a sensitivity of 90.5%, specificity of 87.5%, diagnostic accuracy of 88.5% and F-score of 84.4%, way higher than that of nomogram and RF models (**Table 2**). ML models are sophisticated and it is hard for clinicians to comprehend them, therefore less practiced in clinics (Ou et al., 2020). We have provided a visual illustration of the implemented models to help easily understand the importance of different models and features by clinicians. The results of XGBoost have been explained by LIME plot, which makes it easy to understand the individualized prediction (**Figure 9**).

To the best of our knowledge, this is the first study in the literature to implement and compare XGBoost, RF, and LR model (presented as a nomogram) based on clinical, laboratory data, immune cell and cytokine profiles for the differentiation of critically ill from non-critically ill patients with COVID-19 pneumonia. While global measures such as accuracy are useful, they cannot be used for explaining why a model made a specific prediction. We used LIME plot to explain the outcome of XGBoost model. In addition, cytokine profile and immune cellular data were also evaluated as potential predictors for the severity of COVID-19 pneumonia. Our study still shows limitations and there is room for further improvement. First, it was a retrospective study from a single center. Secondly, the small sample size bears an intrinsic risk of over-fitting though we used two-fold cross-validation as the resampling method to avoid overfitting. Only patients with pneumonia were enrolled, therefore our results may be not applicable to patients without pneumonia. Thirdly, given that the proposed ML method is purely data-driven, our model may vary if applied on different datasets (Yan et al., 2020). Our XGBoost approach needs further model training, validation, and optimization before clinical application because patients in this study were enrolled from a single tertiary referral center. However, the findings are interesting and warrant further research. In future, application of deep learning models on our data would be interesting. Apart from classification of patients suffering from COVID-19, our protocol could be applied to subtype various cancers and could be extrapolated in other viral diseases as well. Amalgamation of more methods, deep learning and unsupervised algorithm comparison could also be interesting. The findings could be useful for doctors in prioritizing patient treatment and be a part of decision support systems to obtain useful predictors and impact clinical outcomes.

In conclusion, comparison stats showed that XGBoost had the highest discriminatory performance for prediction of critically ill patients with COVID-19 pneumonia. The nomogram and visualized interpretation with LIME plot could also be useful in the clinical setting. Additionally, we identified that IL-10 is a useful predictor of critically ill patients with COVID-19 pneumonia and this finding is complemented by previously available literature as well.

## Data Availability Statement

The raw data supporting the conclusions of this article will be made available by the authors, without undue reservation.

## Ethics Statement

This study protocol was approved by the Ethics Committee of the First Affiliated Hospital of Wenzhou Medical University. The committee decided to waive the need for written informed consent from the participants studied in this analysis as the data were analyzed retrospectively and anonymously.

## Author Contributions

WH conceived the study and carried out majority of the work. WH, GC, and JYeP participated in data collection. WH, ZB, XZ, SJ, YL, JYiP, QL, and SY conducted data analysis and drafted the manuscript. TX, ZB, MZ, SF, VT, SS and AG helped to finalize the manuscript. All authors listed have made a substantial, direct, and intellectual contribution to the work and approved it for publication.

## Funding

This work was supported by the Zhejiang Medical and Health Science and Technology Plan Project (Number: 2022KY886), the Wenzhou Science and Technology Bureau (Number: Y2020010), and the Wenzhou Key Technology Breakthrough Program on Prevention and Treatment for COVID-19 Epidemic (Number: ZG2020012).

## Conflict of Interest

The authors declare that the research was conducted in the absence of any commercial or financial relationships that could be construed as a potential conflict of interest.

## Publisher’s Note

All claims expressed in this article are solely those of the authors and do not necessarily represent those of their affiliated organizations, or those of the publisher, the editors and the reviewers. Any product that may be evaluated in this article, or claim that may be made by its manufacturer, is not guaranteed or endorsed by the publisher.

## Supplementary Material

Supplementary Figure 1 Data flow diagram of this study.
