# Supplementary figures and images for: A Comparison of XGBoost, Random Forest, and Nomograph for the Prediction of Disease Severity in Patients With COVID-19 Pneumonia: Implications of Cytokine and Immune Cell Profile
Source: Front Cell Infect Microbiol. 2022 Apr 12;12:819267. doi: 10.3389/fcimb.2022.819267 (PMC9039730; doi:10.3389/fcimb.2022.819267)

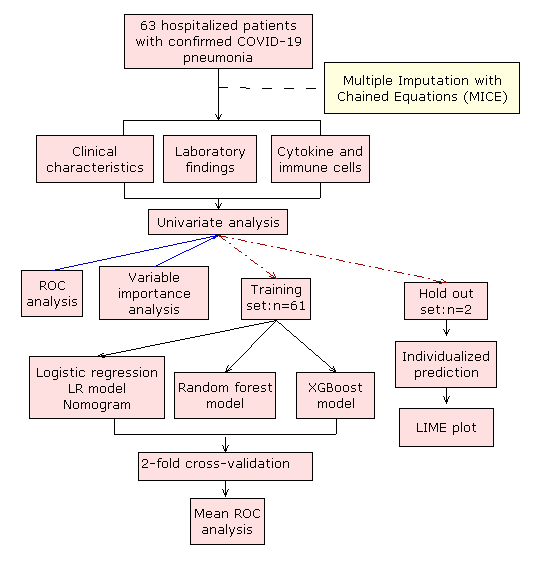

Supplement: Supplementary Figure 1 — Data flow diagram of this study. [file Image_1.tif]
